# Associations of childhood trauma with long-term diseases and alcohol and nicotine use disorders in Czech and Slovak representative samples

**DOI:** 10.1186/s12889-022-14160-2

**Published:** 2022-09-19

**Authors:** Natalia Kascakova, Martina Petrikova, Jana Furstova, Jozef Hasto, Andrea Madarasova Geckova, Peter Tavel

**Affiliations:** 1 Olomouc University Social Health Institute, Palacky University Olomouc, Univerzitní 22, 771 11 Olomouc, Czech Republic; 2 Psychiatric-Psychotherapeutic Outpatient Clinic, Pro mente sana, Heydukova 27, 811 08 Bratislava, Slovakia; 3 Department of Social Work, St. Elizabeth College of Health and Social Work, Palackého 1, 811 02 Bratislava, Slovakia; 4 Department of Health Psychology, Faculty of Medicine, P. J. Safarik University, Trieda SNP 1, 040 11 Kosice, Slovakia

**Keywords:** Childhood trauma, Abuse and neglect, Long-term disease, Nicotine and alcohol use disorders

## Abstract

**Objective:**

The abuse and neglect of a child is a major public health problem with serious psychosocial, health and economic consequences. The aim of this study was to assess the relationship between various types of childhood trauma, selected long-term diseases and alcohol and nicotine use disorder in Czech and Slovak representative samples.

**Methods:**

Data on retrospective reporting about selected long-term diseases, alcohol and nicotine use disorder (CAGE Questionnaire) and childhood maltreatment (Childhood Trauma Questionnaire; CTQ) in two representative samples (Czech sample: *n* = 1800, 48.7% men, mean age 46.61 ± 17.4; Slovak sample: *n* = 1018, 48.7% men, mean age: 46.2 ± 16.6) was collected. Multinomial logistic regression models were used to assess the relationships between childhood maltreatment and long-term diseases.

**Results:**

There is a higher occurrence of some long-term diseases (such as diabetes, obesity, allergy, asthma) and alcohol and nicotine use disorder in the Czech sample; however, in the Slovak sample the associations between child maltreatment and long-term diseases are stronger overall. Emotional abuse predicts the occurrence of all the studied long-term diseases, and the concurrent occurrence of emotional abuse and neglect significantly predicts the reporting of most diseases. All types of childhood trauma were strong predictors of reporting the occurrence of three or more long-term diseases.

**Conclusion:**

The extent of reporting childhood trauma and associations with long-term diseases in the Czech and Slovak population is a challenge for the strengthening of preventive and therapeutic programmes in psychosocial and psychiatric care for children and adolescents to prevent later negative consequences on health.

## Introduction

The abuse and neglect of children is a major public health problem with serious psychosocial, health and economic consequences [1, 2].

Generally, five different types of child abuse and neglect are distinguished: *Emotional abuse* (EA) has been defined as “verbal assaults on a child’s sense of worth or well-being or any humiliating, demeaning or threatening behaviour toward a child by an older person”; *physical abuse* (PA) as “bodily assaults on a child by an older person that posed a risk of, or result in, injury”; *sexual abuse* (SA) as “sexual contact or conduct between a child younger than 18 years of age and an adult or older person”; *emotional neglect* (EN) as “the failure of caretakers to meet children’s basic emotional and psychological needs, including love, belonging, nurturance, and support”; and *physical neglect* (PN) as “the failure of caregivers to provide for a child’s basic physical needs, including food, shelter, clothing, safety, and health care” [3].

Recent analyses of retrospective reports of child maltreatment measured using the Childhood Trauma Questionnaire (CTQ) [3] in Czech and Slovak representative samples indicate the occurrence of emotional abuse in 14.7% and 11.7%, respectively; the occurrence of physical abuse in 11.7% and 11%, respectively; sexual abuse in 7% and 6.7%, respectively; emotional neglect in 18.7% and 17.1%, respectively; and physical neglect in 35.8% and 35.7%, respectively [4–6]. Those were clinically/empirically relevant types of retrospective reports of childhood abuse or neglect according to the clinically derived Walker’s scoring [7].

The pioneering epidemiological study of Felitt et al. [8], which assessed 10 adverse childhood experiences (ACEs) before the age of 18 years, including parental divorce, death of a caregiver, domestic violence, etc., revealed that individuals who have experienced ACEs had an increased risk for several diseases which are leading causes of death worldwide. Many studies in this field followed and were later explored in meta-analyses [1, 9, 10].

A meta-analytic study by Hughes et al. [9] found 11,621 studies concerning the effects of childhood trauma on later health and ultimately revealed from 37 studies comprising 253,719 participants that 4 or more adverse childhood experiences increase the risk of overweight, obesity and diabetes (odds ratio (OR) = 2); moderately increase health-risk behaviour, like smoking or heavy alcohol use, and the risk of cancer, heart and respiratory diseases (OR 2 to 3); highly increase the risk of later risky sexual behaviour, the development of mental illnesses and problematic alcohol use (OR 3 to 6); and robustly elevate the risk of interpersonal and intrapersonal violence (OR more than 7). In a large Dutch population-based study, Noteboom et al. [11] found in a large adult sample (13,489 participants aged 18 to 64) that childhood trauma exposure before 16 years of age predicts the development of many adulthood physical conditions, such as digestive, musculoskeletal and respiratory disorders, with OR ranging from 1.2 to 2.9, even after controlling for sociodemographic and lifestyle factors. Moreover, this Dutch study found indirect associations of childhood trauma with substance use disorders. A German representative study on 2,510 participants above the age of 14 years (average age 48.4 years) [12] showed an increased risk for obesity, cancer, hypertension, myocardial infarction, chronic pulmonal diseases and stroke (OR 1.2 to 1.8) if any kind of maltreatment measured by the CTQ occurred during childhood before the age of 18 years. All of these illnesses were positively associated with higher intensity of maltreatment as well as with an increasing number of experienced maltreatment subtypes.

Clinical practice and results from large representative samples, e.g. Dong et al. [13], reveal that individual subtypes of child maltreatment often co-occur as combined childhood trauma or multiple forms of childhood trauma. There are dose–response relationships between the severity and frequency of childhood trauma and the risk for later disease, while the association between types of childhood trauma and disease outcomes appear to be nonspecific, perhaps because individual types of maltreatment often co-occur [14]. However, a recent Australian prospective study in a large birth cohort revealed that especially emotional abuse and/or emotional neglect are strong predictors for many adverse outcomes in health at age 21 [15, 16].

The above-cited Australian prospective study also revealed the association between child maltreatment and increased onset and persistent smoking [17] and between emotional abuse and neglect and problem alcohol use at age 21 [18]. The link between child maltreatment and later alcohol and nicotine use disorders (AUD, NUD) has also been revealed by large population studies [11, 19] and meta-analytic studies [1, 9].

There is evidence on the cumulative effect of life stressors experienced in childhood and across the life span on worsened health status, with the occurrence of more chronic conditions [20]. This is probably because health conditions associated with early life stress often occur or are aggravated in response to acute stressors in individuals with dysfunctional stress response, which includes changed neurohumoral regulation of the hypothalamic–pituitary–adrenal axis and increased autonomic and inflammatory response [14, 21, 22].

Our aim was to assess the relationship between various childhood trauma types, including concurrent occurrence of emotional abuse and neglect, combined trauma (more than 3 types of trauma) and long-term diseases and alcohol and nicotine use disorder in Czech and Slovak national representative samples, after adjusting for gender and age. We hypothesized that specifically emotional abuse and/or neglect and three or more types of childhood trauma would predict the selected long-term diseases. We also hypothesized that the number of childhood trauma types will be positively associated with the occurrence of three or more long-term diseases. Another hypothesis was that the associations between trauma and the occurrence of long-term diseases would be similar in both the countries.

## Methods

### Research samples and method of data collection

Data from respondents in the Czech population was collected by trained administrators using personal interviews in the respondents’ households during September and November 2016. The selected group of 1800 participants is a representative sample of the population of the Czech Republic over the age of 15 in relation to gender (48.7% men), age composition (age 15 to 88 years old, mean age: 46.41), education and regional affiliation.

In the Slovak population, the data was collected in April 2019 through a professional research agency in the form of personal interviews with trained administrators. The representative Slovak sample consists of 1018 participants, 48.7% men, aged 18 to 85 years (mean age: 46.2). The sample of respondents was compiled on the basis of data from the Statistical Office of the Slovak Republic on the structure of the adult population in terms of gender, age, education, nationality, size of place of living and region of living.

Computer-assisted personal interviewing (CAPI) was used in both samples. CAPI is a method of face-to-face interviewing using a tablet or a computer to record the answers of participants. The advantages of the CAPI method are that a larger set of questionnaires can be collected; it eliminates errors in recording answers, and it significantly saves time by faster processing of the collected data [23].

The sociodemographic characteristics of both the Czech and Slovak samples are listed in Table 1.

**Table 1:** Sociodemographic characteristics

**Characteristic**		CZ (*n* = 1800)		SK (*n* = 1018)	
**Gender**	n (%)				
Male		877 (48.7)		496 (48.7)	
Female		923 (51.3)		522 (51.3)	
**Age**	M (SD)	46.4 (17.4)		46.2 (16.6)	
**Education**	n (%)				
Primary		141 (7.8)		137 (13.5)*	
Skilled operative		442 (24.6)		272 (26.7)	
High school graduate		854 (47.4)*		382 (37.5)	
College		363 (20.2)		227 (22.3)	
**Marital status**	n (%)				
Single		439 (24.4)		225 (22.1)	
Married		929 (51.6)		553 (54.3)	
Divorced		158 (8.8)		72 (7.1)	
Widow/widower		133 (7.4)		78 (7.7)	
Unmarried partner		141 (7.8)		90 (8.8)	
**Child maltreatment (CTQ)**	n (%)	(a)	(b)	(a)	(b)
Emotional abuse		262 (14.6)*	375 (20.8)*	119 (11.7)	161 (15.8)
Physical abuse		210 (11.7)	210 (11.7)	112 (11.0)	112 (11.0)
Sexual abuse		126 (7.0)	189 (10.5)	68 (6.7)	93 (9.1)
Emotional neglect		337 (18.7)	900 (50.0)	174 (17.1)	490 (48.1)
Physical neglect		642 (35.7)	642 (35.7)	364 (35.8)	364 (35.8)
1 type of child maltreatment		398 (22.1)	459 (25.5)	217 (21.3)	260 (25.5)
2 types of childhood maltreatment		192 (10.7)	335 (18.6)	92 (9.0)	178 (18.4)
Emotional abuse + neglect		152 (8.4)	315 (17.5)*	77 (7.6)	142 (13.9)
≥ 3 types of childhood trauma		206 (11.4)	312 (17.3)	111 (10.9)	150 (14.7)
**Long-term diseases**	n (%)				
Hypertension		371 (20.6)*		172 (16.9)	
Ischemic heart disease		70 (3.9)		31 (3.0)	
Obesity		183 (10.2)*		53 (5.2)	
Diabetes		182 (10.1)*		64 (6.3)	
Allergy		364 (20.2)*		124 (12.2)	
Eczema		156 (8.7)*		51 (5.0)	
Asthma		166 (9.2)*		45 (4.4)	
Gastroduodenal ulcer		56 (3.1)		41 (4.0)	
Thyroid gland disease		152 (8.4)*		35 (3.4)	
Migraine		223 (12.4)*		81 (8.0)	
Back pain		631 (35.1)		320 (31.4)	
Arthritis		122 (6.8)		42 (4.1)	
Pain of unclear origin		99 (5.5)		66 (6.5)	
Pelvic pain		68 (3.8)		41 (7.9)	
Depression and anxiety		125 (6.9)*		38 (3.7)	
**Numbers of long-term diseases**	n (%)				
No long-term disease (“healthy”)		406 (22.6)		375 (36.8)*	
1 long-term disease		513 (28.5)		276 (27.1)	
2 long-term diseases		394 (21.9)*		175 (17.2)	
3 and more long-term diseases		487 (27.1)*		192 (18.9)	
**Alcohol use disorder**		184 (10.2)*		69 (6.8)	
**Nicotine use disorder**		295 (16.4)*		126 (12.4)	

Note: *M* Mean, *SD* Standard deviation; (a) occurrence of childhood trauma according to Walker’s clinical cut-off scoring [7] (b) occurrence of childhood trauma according to Bernstein’s cut-off scoring [24], where a low occurrence is already considered to be trauma, *p < 0.05 assessed by Z-test calculator for 2 samples [25], the *p*-value indicates the differences between the two samples

## Measures

### Sociodemographic data

Participants reported gender (male or female), age (continuous), marital status (single, married, divorced, widowed or unmarried partner) and education (primary, skilled operative, high school graduate and college).

### Long-term health complaints

Long-term health difficulties were detected by the item “Do you have any long-lasting disability or disorder? Please, mark all possibilities which are related to you”. Respondents chose from the following list: hypertension, ischemic heart disease, cerebral insult/haemorrhage, diabetes, obesity, chronic pulmonary disease, asthma, cancer, back pain, migraine, pain of unclear origin, pelvic pain – in women, arthritis, dermatitis (eczema), allergy, gastric and duodenal ulcer, inflammatory bowel disease, diseases of thyroid gland, anxiety, depression, or no disease.

### Alcohol use disorder

Alcohol use disorder was detected by questions on alcohol use and using the CAGE questionnaire [26]. The CAGE questionnaire is a quick clinical tool for detecting alcoholism. The questions focus on Cutting down, Annoyance by criticism, Guilty feeling, and Eye-openers. A score of 2 to 3 indicates a high index of suspicion and a score of 4 is virtually diagnostic for alcohol use disorder [27].

### Nicotine use disorder

Nicotine use disorder was also detected by questions on smoking and using the CAGE scale revised for smoking behaviour for assessing nicotine dependence [28]. Two yes answers are positive in screening for nicotine use disorder.

### Childhood trauma

Childhood trauma was measured using the Childhood Trauma Questionnaire (CTQ), a retrospective self-report measuring the severity of five different types of childhood trauma: emotional abuse (EA), physical abuse (PA), sexual abuse (SA), emotional neglect (EN), and physical neglect (PN) [3]. Each subscale has five items rated on a five-point Likert-type scale with response options ranging from (1) never true to (5) very often true. We used Walker’s procedure of severity ratings in the present study [7]. According to Walker’s approach, PA and PN include all cases from “slight to moderate” up to “extreme” childhood trauma (cut-off score 8), and SA and EN include all cases from “moderate to severe” up to “extreme” childhood trauma (8 for SA, 15 for EN). For EA, the cut-off point is in the middle of the “slight to moderate” level (cut-off score 9). The Czech version of the CTQ has been shown to be both reliable and valid. Cronbach’s alpha for the whole questionnaire was 0.92 and for the individual subscales varied from 0.64 to 0.92 [4]. The analysis showed the acceptable reliability and validity of the Slovak version of the CTQ, with Cronbach’s alpha 0.84 and for the individual subscales from 0.64 to 0.94 [5].

The Childhood Trauma Questionnaire and sociodemographic variables were parts of a broader questionnaire battery. Both the Slovak and Czech versions of the CTQ were obtained by means of a back-translation procedure. The original questionnaire was translated from English by two freelance translators and then back into English. The translations were then corrected appropriately.

### Statistical analyses

For statistical analyses, IBM SPSS Statistics software version 21 (IBM Corp., Armonk, New York, NY, USA) was used. The occurrence of various types of abuse and neglect and the selected long-term diseases in both populations were compared by the Z-Test Calculator for two samples [25]. Binary logistic regression models were used to assess the relationships between childhood maltreatment and long-term diseases. The models were assessed univariately, i.e. in each model a specific long-term health condition or alcohol/nicotine use disorder was the outcome (compared to a healthy group or to abstinent persons/non-smokers), and one type of abuse or neglect was considered the predictor. All the univariate models were adjusted for the gender and age of the respondents.

Age and gender were entered into the analyses as covariates. Due to multiple testing, the level of significance was set at α = 0.005. Other levels of *p*-values (*p* < 0.05, *p* < 0.001) are indicated for informative reasons only. The graphs were constructed using the Maple 2020 computer algebra system, displaying the confidence intervals as horizontal line segments.

## Results

**Fig. 1:**
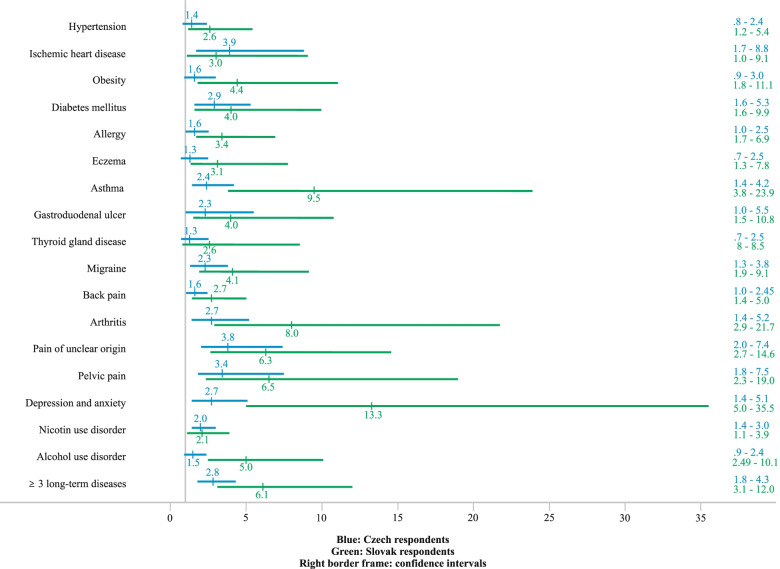
Odds ratios and confidence intervals for the studied long-term diseases in respondents reporting three or more types of childhood trauma

Tables 2, 3, and 4 show the odds ratios (OR) and confidence intervals (CI) adjusted for gender and age for both the Czech and Slovak samples for all examined diseases and disorders. In both samples Emotional abuse was the single statistically significant predictor (with *p* < 0.005) for the majority of the studied diseases. In the Slovak sample Emotional abuse affected the occurrence of all the diseases except Hypertension and Thyroid gland disease with ORs ranging from 2.4 (for Allergy) to 13.8 (for Depression and Anxiety). In the Czech sample the significant ORs of Emotional abuse affecting the occurrence of the long-term diseases were slightly lower, varying from 2.4 (for Allergy) to 4.4 (for Pelvic pain). In the Slovak sample Physical abuse was a statistically significant predictor for six of the individual long-term diseases: Obesity, Allergy, Eczema, Asthma, Pelvic pain, and Depression and Anxiety (with ORs from 2.3 to 5.8). On the other hand, Physical abuse in the Czech sample was a significant predictor only for Diabetes mellitus and Alcohol use disorder (ORs 2.9 and 1.9). The results for Sexual abuse are similar. In the Slovak sample it predicted three of the individual diseases (Obesity, Migraine, and Arthritis with ORs from 3.5 to 4.6), while in the Czech sample it predicted Ischemic heart disease only (OR = 3.3). Emotional neglect predicted the occurrence of Hypertension, Diabetes mellitus, Allergy, Asthma, Gastroduodenal ulcer, Depression and Anxiety and pain-related conditions (Migraine, Back pain, Arthritis, Pain of unclear origin and Pelvic pain) in the Slovak sample (with ORs from 2.4 to 7.8). In the Czech sample Emotional neglect predicted only Migraine, Pain of unclear origin and Depression and Anxiety (with ORs from 1.7 to 2.2). In both samples the concurrent occurrence of Emotional abuse and neglect significantly predicted Diabetes mellitus, Asthma, Migraine, Pain of unclear origin, Pelvic pain, and Depression and Anxiety (with ORs from 2.4 to 4.1 and from 4.6 to 21.2 in the Czech and Slovak sample, respectively) and Alcohol use disorder in the Slovak sample (OR = 4.5). The occurrence of three or more types of childhood trauma predicted the occurrence of most diseases in the Slovak sample (except Ischemic heart disease, Thyroid gland disease and Nicotine use disorder, with ORs from 2.6 to 13.3) and of some diseases in the Czech sample (ORs from 2 for Nicotine use disorder to 3.9 for Ischemic heart disease) (Fig. 1).

**Table 2:** Odds ratios of the occurrence of long-term diseases in association with various types of childhood trauma in Czech and Slovak representative samples adjusted for gender and age

Long-term disease		Czech sample (*n* = 1800)		Slovak sample (*n* = 1018)	
	Type of abuse or neglect	n(%)	OR (95%CI)	n(%)	OR (95%CI)
Hypertension		371 (20.6)		172 (16.9)	
	Emotional abuse		1.8 (1.1–3.0)*		2.3 (1.1–4.8)*
	Physical abuse		1.2 (0.7–2.0)		1.7 (0.9–3.5)
	Sexual abuse		1.2 (0.6–2.2)		1.0 (0.4–2.7)
	Emotional neglect		1.1 (0.7–1.6)		2.4 (1.3–4.4)**
	Physical neglect		1.6 (1.2–2.3)**		1.5 (0.9–2.4)
	Emotional abuse + neglect		1.6 (0.8–2.9)		2.7 (1.1–6.5)*
	≥ 3 types of trauma		1.4 (0.8–2.4)		2.6 (1.2–5.4)**
Ischemic heart disease		70 (3.9)		31 (3.0)	
	Emotional abuse		3.1 (1.4–6.8)**		4.9 (1.7–14.1)**
	Physical abuse		2.4 (1.1–5.2)*		1.0 (0.3–3.9)
	Sexual abuse		3.3 (1.4–7.9)**		1.7 (0.3–8.4)
	Emotional neglect		1.8 (0.9–3.4)		2.0 (0.7–5.9)
	Physical neglect		2.3 (1.3–4.1)**		1.3 (0.6–2.9)
	Emotional abuse + neglect		2.9 (1.1–7.5)*		5.3 (1.5–18.7)*
	≥ 3 types of trauma		3.9 (1.7–8.8)***		3.0 (1.0–9.1)*
Obesity		183 (10.2)		53 (5.2)	
	Emotional abuse		3.4 (2.0–5.7)***		3.5 (1.5–8.2)**
	Physical abuse		1.5 (0.8–2.7)		3.0 (1.3–6.7)**
	Sexual abuse		1.5 (0.8–3.0)		4.2 (1.6–10.8)**
	Emotional neglect		1.3 (0.8–2.0)		2.4 (1.1–5.4)*
	Physical neglect		1.3 (0.9–1.9)		2.6 (1.4–4.9)*
	Emotional abuse + neglect		2.4 (1.2–4.6)*		3.9 (1.4–10.9)*
	≥ 3 types of trauma		1.6 (0.9–3.0)		4.4 (1.8–11.1)***
Diabetes mellitus		182 (10.1)		64 (6.3)	
	Emotional abuse		2.8 (1.6–5.0)***		4.4 (1.8–10.5)***
	Physical abuse		2.9 (1.6–5.0)***		1.2 (0.4–3.2)
	Sexual abuse		2.2 (1.1–4.4)*		1.1 (0.3–4.4)
	Emotional neglect		1.7 (1.1–2.7)*		4.6 (2.2–9.6)***
	Physical neglect		2.0 (1.4–3.0)***		2.0 (1.1–3.7)*
	Emotional abuse + neglect		2.8 (1.4–5.6)**		8.3 (3.0–22.2)***
	≥ 3 types of trauma		2.9 (1.6–5.3)***		4.0 (1.6–9.9)**
Allergy		364 (20.2)		124 (12.2)	
	Emotional abuse		2.4 (1.5–3.4)***		2.4 (1.2–4.6)**
	Physical abuse		1.6 (1.0–2.6)*		2.3 (1.2–4.4)**
	Sexual abuse		1.3 (0.8–2.3)		2.5 (1.2–5.2)*
	Emotional neglect		1.2 (0.8–1.7)		2.5 (1.4–4.5)***
	Physical neglect		1.2 (0.9–1.6)		2.5 (1.6–3.8)***
	Emotional abuse + neglect		1.9 (1.1–3.2)*		2.6 (1.2–5.9)*
	≥ 3 types of trauma		1.6 (1.0–2.5)		3.4 (1.7–6.9)***
Eczema		156 (8.7)		51 (5.0)	
	Emotional abuse		1.9 (1.1–3.3)*		3.8 (1.7–8.5)**
	Physical abuse		1.5 (0.8–2.8)		3.1 (1.4–7.0)**
	Sexual abuse		1.3 (0.7–2.8)		2.4 (0.8–6.8)
	Emotional neglect		1.1 (0.7–1.8)		1.6 (0.7–3.8)
	Physical neglect		1.3 (0.9–1.9)		1.4 (0.8–2.7)
	Emotional abuse + neglect		1.3 (0.6–2.7)		1.8 (0.5–6.8)
	≥ 3 types of trauma		1.3 (0.7–2.5)		3.1 (1.3–7.8)**

*Note*: ***p < 0.001, ** *p* < 0.005, *p < 0.05; Boldface values denote *p* < 0.005; The reference group is made up of respondents without long-term disease (“healthy”). Individual types of child maltreatment (“trauma”) are scored according to Walker’s clinical cut-off scoring [7]

**Table 3:** Odds ratios of the occurrence of long-term diseases in association with various types of childhood trauma in Czech and Slovak representative samples adjusted for gender and age

Long-term disease		Czech Sample (*n* = 1800)		Slovak sample (*n* = 1018)	
	Type of abuse or neglect	n(%)	OR (95%CI)	n(%)	OR (95%CI)
Asthma		166 (9.2)		45 (4.4)	
	Emotional abuse		3.4 (2.0–5.6)***		6.6 (2.9–15.3)***
	Physical abuse		1.9 (1.1–3.3)*		5.8 (2.6–13.0)***
	Sexual abuse		1.9 (1.0–3.6)*		2.3 (0.7–7.7)
	Emotional neglect		1.6 (1.0–2.5)*		4.8 (2.3–10.4)***
	Physical neglect		1.5 (1.0–2.3)*		3.3 (1.7–6.4)***
	Emotional abuse + neglect		3.0 (1.6–5.6)***		7.5 (2.7–21.1)***
	≥ 3 types of trauma		2.4 (1.4–4.2)**		9.5 (3.8–23.9)***
Gastroduodenal ulcer		56 (3.1)		41 (4.0)	
	Emotional abuse		3.3 (1.5–7.1)**		3.4 (1.3–8.6)**
	Physical abuse		2.4 (1.1–5.3)*		1.9 (0.7–5.1)
	Sexual abuse		1.3 (0.4–4.0)		1.8 (0.5–6.7)
	Emotional neglect		1.3 (0.6–2.6)		4.1 (1.9–9.0)***
	Physical neglect		1.6 (0.9–2.9)		1.9 (1.0–3.8)
	Emotional abuse + neglect		2.1 (0.7–5.9)		5.4 (1.9–15.5)**
	≥ 3 types of trauma		2.3 (1.0–5.5)*		4.0 (1.5–10.8)**
Thyroid gland disease		152 (8.4)		35 (3.4)	
	Emotional abuse		2.1 (1.2–3.7)*		3.0 (1.0–8.7)*
	Physical abuse		1.2 (0.7–2.4)		0.9 (0.2–4.1)
	Sexual abuse		2.1 (1.1–4.0)*		0.7 (0.1–5.4)
	Emotional neglect		0.9 (0.6–1.6)		2.3 (0.9–6.1)
	Physical neglect		1.1 (0.7–1.7)		1.6 (0.7–3.4)
	Emotional abuse + neglect		1.8 (0.9–3.6)		2.9 (0.8–11.3)
	≥ 3 types of trauma		1.3 (0.7–2.5)		2.6 (0.8–8.5)
Migraine		223 (12.4)		81 (8.0)	
	Emotional abuse		2.8 (1.7–4.5)***		3.9 (1.9–7.9)***
	Physical abuse		1.8 (1.1–3.0)*		2.2 (1.0–4.8)*
	Sexual abuse		1.6 (0.9–3.0)		3.5 (1.5–8.1)**
	Emotional neglect		1.7 (1.2–2.6)**		3.2 (1.7–6.2)***
	Physical neglect		1.4 (0.1–2.0)*		2.3 (1.4–3.9)***
	Emotional abuse + neglect		2.4 (1.3–4.5)**		4.6 (2.0–10.8)***
	≥ 3 types of trauma		2.3 (1.3–3.8)**		4.1 (1.9–9.1)***
Back pain		631 (35.1)		320 (31.4)	
	Emotional abuse		2.5 (1.7–3.8)***		3.0 (1.7–5.2)***
	Physical abuse		1.6 (1.0–2.4)*		1.4 (0.8–2.6)
	Sexual abuse		0.9 (0.5–1.6)		1.7 (0.8–3.5)
	Emotional neglect		1.3 (0.9–1.8)		2.4 (1.5–3.9)***
	Physical neglect		1.2 (0.9–1.7)		1.9 (1.4–2.8)***
	Emotional abuse + neglect		1.9 (1.2–3.3)*		3.1 (1.5–6.2)***
	≥ 3 types of trauma		1.6 (1.0–2.45)*		2.7 (1.4–5.0)**
Arthritis		122 (6.8)		42 (4.1)	
	Emotional abuse		2.2 (1.1–4.3)*		4.2 (1.5–11.2)**
	Physical abuse		1.9 (1.0–3.8)*		1.2 (1.1–1.2)
	Sexual abuse		1.7 (0.7–3.8)		4.6 (1.5–13.9)**
	Emotional neglect		1.9 (1.1–3.1)*		5.3 (2.4–11.9)***
	Physical neglect		1.8 (1.1–2.8)*		4.1 (2.0–8.5)***
	Emotional abuse + neglect		1.9 (1.0–3.3)*		7.1 (2.3–22.1)***
	≥ 3 types of trauma		2.7 (1.4–5.2)**		8.0 (2.9–21.7)***

*Note*: ***p < 0.001, ** *p* < 0.005, *p < 0.05; Boldface values denote *p* < 0.005; The reference group are respondents without long-term disease (“healthy”). Individual types of child maltreatment (“trauma”) are scored according to Walker’s clinical cut-off scoring [7]

**Table 4:** Odds ratios of the occurrence of long-term diseases in association with various types of childhood trauma in Czech and Slovak representative samples adjusted for gender and age

Long-term disease		Czech sample (*n* = 1800)		Slovak sample (*n* = 1018)	
	Type of abuse or neglect	n(%)	OR (95%CI)	n(%)	OR (95%CI)
Pain of unclear origin		99 (5.5)		66 (6.5)	
	Emotional abuse		4.2 (2.3–7.7)***		6.0 (2.8–13.0)***
	Physical abuse		2.3 (1.2–4.4)*		2.1 (0.9–4.8)
	Sexual abuse		1.5 (0.6–3.5)		1.6 (0.5–5.1
	Emotional neglect		2.2 (1.3–3.7)**		5.0 (2.6–9.9)***
	Physical neglect		2.1 (1.3–3.3)**		2.3 (1.3–4.1)**
	Emotional abuse + neglect		4.1 (2.0–8.5)***		6.9 (2.6–18.0)***
	≥ 3 types of trauma		3.8 (2.0–7.4)*		6.3 (2.7–14.6)***
Pelvic pain		68 (3.8)		41 (7.9)	
	Emotional abuse		4.4 (2.4–8.4)***		4.5 (1.7–12.0)**
	Physical abuse		2.2 (1.0–4.6)*		4.3 (1.5–11.9)**
	Sexual abuse		2.0 (0.8–4.7)		1.6 (0.4–6.3)
	Emotional neglect		1.9 (1.0–3.5)*		4.7 (1.9–11.6)***
	Physical neglect		2.0 (1.2–3.4)*		2.5 (1.3–4.8)**
	Emotional abuse + neglect		3.8 (1.7–8.5)***		5.7 (1.7–19.1)**
	≥ 3 types of trauma		3.4 (1.8–7.5)***		6.5 (2.3–19.0)***
Depression and anxiety		125 (6.9)		38 (3.7)	
	Emotional abuse		3.8 (2.2–6.5)***		13.8 (5.7–33.2)***
	Physical abuse		0.9 (0.4–1.9)		3.8 (1.5–9.7)**
	Sexual abuse		1.6 (0.7–3.4)		4.0 (1.3–12.8)*
	Emotional neglect		2.2 (1.4–3.6)***		7.8 (3.5–17.4)***
	Physical neglect		2.4 (1.6–3.7)***		3.8 (1.8–7.9)***
	Emotional abuse + neglect		3.1 (1.5–6.4)**		21.2 (7.6–59.3)***
	≥ 3 types of trauma		2.7 (1.4–5.1)**		13.3 (5.0–35.5)***
More than 3 long-term diseases		487 (27.1)		192 (18.9)	
	Emotional abuse		3.9 (2.5–6.0)***		5.5 (3.0–10.8)***
	Physical abuse		2.2 (1.4–3.4)***		2.7 (1.4–5.2)**
	Sexual abuse		1.8 (1.1–3.2)*		2.7 (1.2–5.9)*
	Emotional neglect		1.7 (1.2–2.4)**		3.9 (2.2–6.7)***
	Physical neglect		1.9 (1.4–2.5)***		2.6 (1.7–4.0)***
	Emotional abuse + neglect		2.9 (1.7–5.0)***		6.9 (3.2–14.9)***
	≥ 3 types of trauma		2.8 (1.8–4.3)***		6.1 (3.1–12.0)***
Nicotine use disorder		295 (16.4)		126 (12.4)	
	Emotional abuse		1.8 (1.8–2.5)***		2.4 (1.4–4.2)**
	Physical abuse		1.6 (1.1–2.4)*		1.9 (1.0–3.5)*
	Sexual abuse		1.6 (1.0–2.6)*		2.0 (1.0–4.1)*
	Emotional neglect		1.2 (0.9–1.7)		1.1 (0.7–2.0)
	Physical neglect		1.5 (1.2–2.0)**		1.2 (0.8–1.8)
	Emotional abuse + neglect		1.8 (1.2–2.8)*		2.0 (1.0–4.2)
	≥ 3 types of trauma		2.0 (1.4–3.0)***		2.1 (1.1–3.9)*
Alcohol use disorder		184 (10.2)		69 (6.8)	
	Emotional abuse		3.0 (0.8–5.9)*		6.2 (3.2–11.9)***
	Physical abuse		1.9 (1.2–2.8)**		4.3 (2.2–8.5)***
	Sexual abuse		1.4 (0.9–2.2)		5.0 (2.3–11.0)***
	Emotional neglect		1.0 (0.6–1.9)		1.4 (0.7–2.6)
	Physical neglect		1.3 (0.9–2.0)		1.7 (1.0–3.0)*
	Emotional abuse + neglect		1.0 (0.7–1.4)		4.5 (3.0–6.8)***
	≥ 3 types of trauma		1.5 (0.9–2.4)		5.0 (2.5–10.1)***

*Note*: ***p < 0.001, ** *p* < 0.005, *p < 0.05; Boldface values denote *p* < 0.005; The reference group are respondents without long-term disease (“healthy”). Individual types of child maltreatment (“trauma”) are scored according to Walker’s clinical cut-off scoring [7]

**Fig. 2:**
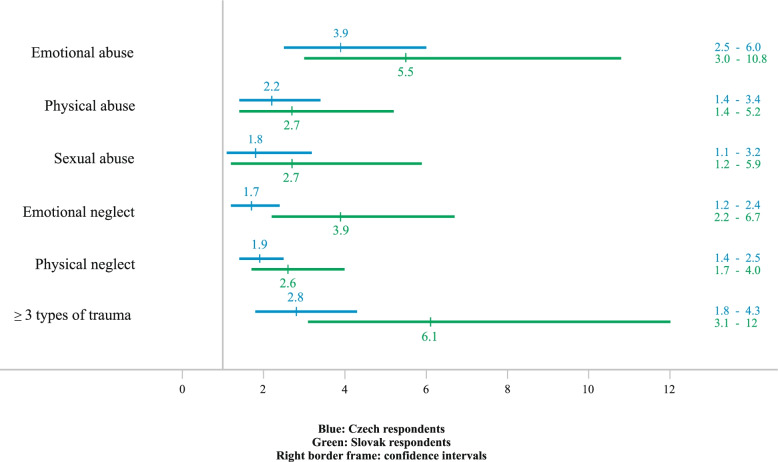
Odds ratios and confidence intervals for occurrence of 3 or more long-term diseases in respondents reporting individual types of childhood trauma and 3 or more types of childhood trauma

In respondents with three or more long-term diseases, all the types of childhood trauma were strong predictors, except Sexual abuse (ORs 1.9 to 3.9 and 2.6 to 5.5 in the Czech and Slovak sample, respectively). In the Czech sample there is a higher occurrence of some diseases (e.g. Obesity, Diabetes mellitus, Allergy). In the Slovak sample, however, there were overall stronger associations (i.e. systematically higher ORs) of those abuse and neglect variables that are significant predictors of long-term diseases (Fig. 2).

The differing strength of predictors between the samples was even more apparent in the predictors of the Nicotine and Alcohol use disorders. For example, though significant for both samples (*p* < 0.005), the OR for Physical abuse affecting Alcohol use disorder was approximately twice as large for the Slovak sample (OR = 1.9 and OR = 4.3 for Czech and Slovak samples, respectively). Overall, in the Czech sample the neglects and abuses were more associated with Nicotine use disorder, while in the Slovak sample the neglects and abuses were more associated with the Alcohol use disorder. For instance, Emotional abuse was the only significant predictor for both Nicotine and Alcohol use disorders in the Slovak sample, with the ORs being almost three-times larger for Alcohol use disorder (OR = 2.4 and OR = 6.2, respectively).

## Discussion

This study was the first to investigate the associations between retrospectively reported childhood trauma and later long-term diseases and alcohol and nicotine use disorder in adulthood in representative samples from the Czech Republic and Slovakia. The analysis revealed that emotional abuse is a significant predictor for most of the studied long-term diseases, as well as alcohol and nicotine use disorders. Although in the Czech sample there is higher percentage of occurrence of long-term diseases, such as diabetes, obesity, eczema, allergy, alcohol and nicotine use disorders, in the Slovak sample the associations between child maltreatment and studied long-term diseases are stronger overall.

In our study, emotional abuse was a significant predictor for most of the studied long-term diseases. Emotional abuse, including parental verbal abuse, has received less attention in research than the more studied and visible physical and sexual abuse, but its negative impact is unquestionable. Devaluing and hurtful words can have a profoundly negative impact on self-image and self-esteem and, moreover, there is evidence that emotional abuse also has its neurobiological correlate in the brain [29]. In a study of Carpenter et al. [30], a history of self-reported childhood emotional abuse significantly diminished cortisol response, independently of the effects of other types of childhood maltreatment. It is important to note that some individuals deny experiencing emotional abuse, although they describe incidents that could be interpreted as such. In a mixed quantitative–qualitative study [31] this group of respondents, denying or not-recognising emotional abuse, had poorer health in adulthood, similarly to respondents recognising and reporting emotional abuse.

According to the results of the present study, physical abuse predicted the occurrence of obesity, allergy, eczema, asthma, pelvic pain, depression and anxiety and alcohol use disorder in the Slovak sample, and only the occurrence of diabetes mellitus and alcohol use disorder in the Czech sample. In a population-based study of middle-aged men and women [32] the physical abuse experienced in childhood predicted worse mental and physical health decades after the abuse. A study by Springer [33] assessing four life course pathways between childhood physical abuse and midlife physical health revealed that health behaviour (such as obesity, drinking and smoking) and mental health problems (such as depression, anxiety) may be crucial links between early childhood physical abuse and midlife physical health.

Differences between the two national samples could also be observed for sexual abuse as a predictor; while in the Slovak sample it predicted the occurrence of obesity, migraine, arthritis and alcohol use disorder, in the Czech sample sexual abuse predicted the occurrence of ischemic heart disease only. According to meta-analytic studies [32, 34], survivors of childhood sexual abuse are at significant risk of a wide range of health difficulties, including obesity, pain-related conditions, cardiopulmonary symptoms, gastrointestinal health and gynaecologic health; moreover, sexual abuse is considered to be a nonspecific risk factor for later psychopathology. We think that in the case of retrospectively reported sexual abuse, there is still tendency to underreport it due to secrecy and stigma, and the real occurrence could be higher.

Most research in the field of child maltreatment has focused on abuse (mainly on physical and sexual abuse), while studying neglect (both emotional and physical) has long been omitted [35]. Emotional neglect is qualitatively different from abuse, because it is associated with a lack of appropriate stimulation and interaction, and like emotional abuse, it is not as visible and well recognized as physical abuse. Lack of emotional nurturing in childhood has been shown to negatively impact the reward system in the brain and reduce the amount of oxytocin receptors in the brain [36]. Reduced reward activation may predict risk for depression, addiction and other psychopathologies [15]. In the present study, emotional neglect predicted the occurrence of all long-term pain-related conditions in the Slovak sample, whereas in the Czech sample it predicted only migraine and pain of unclear origin. In both samples it strongly predicted depression and anxiety.

The questions for detecting physical neglect in the CTQ comprise not only poverty, lack of food and clean clothing but also dysfunctional households with caregivers unable to take appropriate care of a child because of alcoholism, drug use or mental illness. Data from a longitudinal Minnesota study of Risk and Adaptation showed that physical neglect, but not physical or sexual abuse, predicted all three studied health outcomes, including the biomarkers of the cardiometabolic risk, self-reported quality of health and a number of health problems [37]. In the present study, physical neglect was a strong predictor for some pain-related diseases in the Slovak sample and for cardiometabolic diseases (such as hypertension, obesity and diabetes mellitus) in the Czech sample.

Importantly, the co-occurrence of emotional abuse and neglect was a strong predictor for the occurrence of depression and anxiety, more pain-related long-term diseases, diabetes mellitus and asthma in the Czech and Slovak populations and for allergy, gastroduodenal ulcer and alcohol use disorder in the Slovak sample. A large prospective study of Kisely et al. [16] found concurrent emotional abuse and neglect to be the strongest predictors for later anxiety and depressive disorders in adulthood (ORs 2.3 and 2.8, respectively). Moreover, the same prospective study found that emotional abuse and neglect were associated with the greatest numbers of adverse outcomes in the cognitive, psychological, addiction, sexual and physical health outcomes [15]. Interestingly, it seems that results for concurrent emotional abuse and neglect were primarily driven by emotional abuse in most diseases, except diabetes mellitus and gastroduodenal ulcer in the Slovak sample. A recent study found strong associations between moderate to severe childhood neglect and stronger psychological stress response in patients with diabetes mellitus [38]. The associations between possible emotional neglect and gastroduodenal ulcer have already been described by the father of psychosomatic medicine, Franz Alexander, who wrote that “ulcer patients cannot freely gratify their dependent needs because accepting help from others mobilizes shame and guilt” [39].

Individual types of childhood trauma often co-occur in a combination of three or more types of abuse and neglect as combined or multiple childhood trauma [13]. In our research samples the following occurrences of three or more trauma types, according to different types of scoring, were found: in the Czech sample in 11.4% according to Walker (clinically relevant scoring) [7], and in 17.3% according to Bernstein (when already low occurrence is considered to be trauma) [24]; in the Slovak sample in 10.9% and in 14.7%, respectively. In a German representative sample the occurrence of three or more types of childhood trauma was found in 16.6% [40], and in a study in the Netherlands across a 5-year period, the prevalence was 13.0% [41]. We found interesting that the occurrence of three or more trauma types did not predict the occurrence of long-term diseases as strongly and/or significantly, while some individual types of trauma or concurrent emotional abuse and neglect were stronger predictors for diseases. This may be due to the “dose-dependent effect” characterised by greater intensity and frequency of some individual types of maltreatment and its greater effect on heath [19]. However, in the present study, we have not assessed the severity of maltreatment in association with diseases. In the case of concurrent emotional abuse and neglect, the stronger effect on health might be explained by a more profound effect on neurodevelopmental processes. A recent neurobiological study by Puetz et al. [42] revealed that participants who experienced a combination of abuse and neglect showed a hypoactive pattern of neural response in amygdala, with hypocortisolism and a spatially distributed pattern of reduced neural activation in a range of brain areas. Although hypocortisolism can be adaptive in the short-term perspective and reflects the processes of how the organism tries to adjust to a persistent stressful environment in terms of the allostatic load model [43], from a long-term perspective it poses a major threat to healthy development [44]. Another important perspective is developmental programming reflecting the concept of sensitive periods of plasticity in brain development that were identified for various brain areas, various ages and gender differently [45].

The differences between the Czech and Slovak samples were apparent in the case of nicotine and alcohol use disorders (NUD and AUD). Although the occurrence of AUD was significantly higher in the Czech Republic, the magnitude of the effect of physical abuse on AUD was approximately twice as large for the Slovak sample. OECD statistics [46] show that the consumption of alcohol in the Czech Republic was 11.7 L per capita in 2016 (the time of data collection), whereas in Slovakia it was 10.3 L per capita in 2019. The Czech Republic has a long tradition of beer drinking: therefore, we assume that in the case of AUD in the Czech Republic there might be more “traditional drinkers” and a smaller percentage of “symptomatic drinkers” related to childhood maltreatment as in the Slovak sample, where all types of abuse and emotional abuse with neglect were strong predictors of AUD. A prospective study showed that emotional abuse was associated with heavy drinking and emotional neglect with AUD at 21 years of age [18], and emotional and physical abuse predicted AUD at 30 years of age [47]. According to the National Epidemiologic Survey on Alcohol and Related Conditions [48], all types of abuse and physical neglect predicted alcohol and nicotine addiction, also controlled for other childhood adversities. Tobacco use is a major risk factor for cardiovascular and respiratory diseases, different types or subtypes of cancer, and many other impairing health conditions. Alcohol consumption has many negative health and social consequences related to intoxication (e.g. accidents, suicide attempts, injuries) or to use disorder (e.g. liver disease, hypertension, cancer). Alcohol and tobacco use are therefore major socioeconomic problems and are the subject of national preventive programmes [49].

In respondents with three or more long-term diseases, all types of childhood trauma, except sexual abuse, were strong predictors for reporting them. In a study of 97 internal medicine outpatients, the number of childhood trauma types was found to be associated with the number of psychophysiological disorders and with the length of medical disability [50]. Another study of a Canadian community sample [51] revealed that the more childhood traumatic events participants experienced, the more chronic conditions they reported. Disorders related to early stress often occur in comorbidity and manifest or worsen in response to acute stress. Early life stress induces “biological scars” at the level of stress regulatory systems which promote the pathophysiology of various disorders in an interaction with genetics, epigenetics and the environment [14, 52].

One of the aims of this study was to compare the two countries in associations between child maltreatment and health outcomes. The Czech and Slovak Republics formed one country – “Czechoslovakia” – from 1918 to 1992. They separated in 1993. Economic development in the Czech Republic and Slovakia has been very different in recent decades. For example, in 1998 the GNI (gross national income) per capita in the Czech Republic was 72% of the EU average, while in the Slovak Republic it was only 52%. Up to 2013 the economy of the Slovak Republic grew rapidly and approached that of the Czech Republic, but since then the Slovak economy has stagnated and the Czech economy has continued to grow [53]. The life expectancy in the Czech Republic, 75.1 years in 2000 and 78.3 in 2020, is higher than that of Slovakia, with 73.4 years in 2000 and 77.0 in 2020 [53]. The stronger relationship between reported childhood trauma and long-term diseases in the Slovak sample can be explained by the underfinanced and less available psychosocial and psychiatric care in children and adolescents, also in the terms of prevention strategies, compared to other countries, including the Czech Republic [53, 54].

The reason for the higher occurrence of self-reported long-term diseases in Czech inhabitants compared to Slovaks could be better preventive screening of long-term diseases in the Czech Republic. On the other hand, the higher occurrence of some diseases in the Czech Republic (mainly cardiovascular and metabolic diseases) could be related to higher proportions of overweight/obese population, higher alcohol and tobacco consumption and higher use of vaping products in the Czech Republic compared to Slovakia [46]. Another reason might be the dissimilar stratification of the urban and rural populations in both countries. At the time of data collection, 21.9% of inhabitants of the Czech Republic lived in large cities (with more than 100 thousand people) [55], whereas in Slovakia this was only 12.4% [56]. This difference between the countries has been apparent for decades [57], as well as in the representation of the population in the small villages (fewer than 2 thousand inhabitants) – 26.9% in the Czech Republic and 30% in the Slovak Republic. There is no available resource about the occurrence of chronic or long-term diseases in the population according to place or size of living, but a Scottish study comparing rural areas and cities in health outcomes found that people living in remote small towns had a lower risk of hospital admission for chronic disease, and those in very remote rural areas had lower mortality, both compared with those living in primary cities [58]. From the nationality point of view, the Czech Republic was for decades relatively homogenous, with the largest minority being the Roma minority with 2.2%, according to the European Roma Rights Centre [59]. In contrast with the Czech Republic, Slovakia has a large Hungarian minority (22% before 1950, 11.3% in 1980, 7.75% in 2021), Ukrainian minority (0.8% in 1980, 0.17% in 2021) and Ruthenian minority (0.44% in 2021) [60, 61]. Data on the Roma minority are very different; according to the census, the representation of Roma inhabitants is 1.23% in 2021 [61], though according to the Atlas of Roma Communities, 8.06% of inhabitants of Slovakia are Roma [62].

All the associations in the present study were adjusted for gender and age of the respondents; however, other socioeconomic factors could have a confounding effect on childhood trauma, as well. Unfortunately, our study did not include data on the education and socioeconomic status of the respondents’ parents. Research indicates that socioeconomic status (SES) may be transmitted across generations and is related to individual health. Adults with higher educational attainment have better health compared to their less educated peers [63, 64]. Moreover, low SES among parents has been identified as a risk factor for childhood maltreatment, which in turn leads to financial and employment-related difficulties in adulthood [65]. In previous studies in Czech and Slovak representative samples, participants with lower achieved education reported significantly higher occurrence of abuse and neglect in childhood compared to participants with higher achieved education [4, 5]. Childhood abuse or neglect occurs in certain socioeconomic backgrounds, and both might have an impact on the upward or downward trajectory of an individual through socioeconomic stratification, resulting in certain health consequences. The patterns of associations between childhood trauma and the studied diseases might differ if the effects of childhood trauma had been examined over and above the effects of socioeconomic status on the diseases.

### Strengths and limitations

The strength of this study is that it is based on two representative national samples. The same research designs and the same methods used allowed for a comparison of the results.

This study is cross-sectional and associations between childhood maltreatment and health outcomes need to be interpreted with caution. On the other hand, when we know the time causality of events and the effects, the neurodevelopmental processes and mechanisms linking childhood trauma and negative health consequences, we can also presuppose causality between childhood trauma and health outcomes from cross-sectional data. Another limitation could be that data about childhood trauma are retrospectively recalled and thus potentially biased. Studies show a tendency to underreport childhood maltreatment when asked retrospectively [66], and the real frequencies could be higher. Data on long-term diseases were based on self-report and thus could be medically imprecise. On the other hand, a self-reported checklist of long-term diseases has been commonly used as a valid option in research [67]. Finally, another limitation could be confounding factors. The results were controlled for gender and age, but not for education or socioeconomic status of the respondents’ parents or the participants themselves. As stated earlier, the patterns of associations between childhood trauma and the studied diseases might differ if the effects of childhood trauma had been examined over and above the effects of socioeconomic status on the diseases.

## Conclusion

The extent of reporting childhood trauma and associations with long-term diseases, alcohol and nicotine use disorder in the Czech and Slovak populations is a challenge for the strengthening of preventive and therapeutic programmes in the psychosocial and psychiatric care of children and adolescents to prevent long-term negative health consequences later in life. Emotional abuse and emotional neglect, as less visible types of maltreatment, deserve attention in recognising their signs and in developing suitable interventions for reversing health problems in later life.

Knowledge about a potential developmental link with the poor health outcomes may motivate doctors to address this and, when needed, to refer a patient to appropriate psychological/psychiatric treatment. Because childhood maltreatment interferes with the healthy development of a child and possesses a risk for later health problems, intervention preventive programmes in childhood can serve as very useful strategies for healthy development, from the neurobiological aspect as well as from the economical aspect [68]. A 27-years prospective birth cohort study showed that higher levels of economic disadvantage, poor parental mental health, substance use and social instability were strongly associated with increased risk of child maltreatment [69]. Therefore, supporting at-risk parents as early as during pregnancy and then in a child’s early infancy, childhood and adolescence should be one of the priorities in national health policies.

## Data Availability

The datasets generated and analysed during the current study are not publicly available due to Czech and Slovak legislation but are available from the corresponding author on reasonable request.
